# How inter-ethnic relations are reproduced through everyday social practices: A perspective from the Javanese nasi pecel vendors in Mataraman cities

**DOI:** 10.1016/j.heliyon.2024.e30843

**Published:** 2024-05-09

**Authors:** Jony Eko Yulianto, Gabriela Laras Dewi Swastika, Indra Yohanes Kiling

**Affiliations:** a School of Psychology, Universitas Ciputra Surabaya, Surabaya, 60219, Indonesia; b School of Communication and Media Business, Universitas Ciputra Surabaya, 60219, Indonesia; c Department of Psychology, Universitas Nusa Cendana, Kupang, 85001, Indonesia

**Keywords:** Chinese Indonesians, Indonesia, Inter-ethnic relations, Javanese, Nasi pecel, Solidarity

## Abstract

There is growing scholarship on how ethnic groups with historical tensions recover and manage to build harmonious relationships. However, detailed accounts of the lived experiences of such relations are limited. We seek to address this gap by exploring everyday inter-ethnic relations between Javanese and Chinese Indonesians in Indonesia as exemplified in the practice of selling nasi pecel, the traditional food of Mataraman cities in East Java. Our eight-week fieldwork involved 30 nasi pecel sellers in the four cities of Madiun, Nganjuk, Kediri, and Jombang through go-alongs and subsequent photo-elicitation interviews. Our engagements with the sellers have enabled us to generate a large body of empirical materials comprising 35 interviews and over 200 photographs. In the roles of bricoleurs, we then worked abductively to make sense of the empirical materials generated to build case studies of six sellers which resonated with the stories of the other 24 nasi pecel sellers in the study. We focused on the centrality of the seemingly mundane everyday practices of selling nasi pecel in (re)producing inter-ethnic interactions between the Javanese nasi pecel sellers and the Chinese Indonesian landowners. The everyday interactions for purposes such as accessing electricity and water and serving the customers which have been enacted every day for decades build spaces for inter-ethnic friendship and solidarity. We discuss how such inter-ethnic relations are vital in Indonesian society by emplacing such phenomenon within the broader socio-historical context of Chinese Indonesian and Javanese inter-ethnic relations, which are often framed as adversarial.

## 1 Introduction

Scholars investigating inter-ethnic conflicts generally study the nature of the conflicts within the context of national-level politics [1,2]. However, many ethnic groups recover from conflict and live side-by-side harmoniously and the ways in which these groups conduct their everyday lives need to be explored [3]. Subsequently, contemporary research has begun to explore inter-ethnic cooperation in communities recovering from ethnic violence [[4], [5], [6], [7]]. For example, scholars have recently investigated how inter-ethnic dating has enabled people in some countries to overcome structural segregation and inter-ethnic division [4]. In the context of multicultural countries, scholars have also documented how inter-ethnic marriages are common and how partners build liminal spaces for themselves to navigate the tensions that come from their memberships of conflicting ethnic groups [7]. This research provides insights into inter-ethnic relations that are more complex, dynamic, and multifaceted than the literature often suggests. In this research, we document how two ethnic groups with prolonged historical tensions produce and reproduce inter-ethnic collaboration through the practices of selling traditional food [6]. In other words, we approach the practice of selling nasi pecel as a site for everyday inter-group interactions between Javanese nasi pecel sellers and Chinese Indonesian landowners.

In this research, we were interested in exploring inter-ethnic relations between the Javanese nasi pecel sellers and the Chinese Indonesian landowners in four Matamaran cities. We chose this focus because a strong narrative of collaborative inter-ethnic interactions emerged from the everyday lived experiences of the nasi pecel sellers in the four cities, where the Javanese nasi pecel sellers occupy the spaces in front of the Chinese Indonesian-owned shops on the main roads of the cities including Cokroaminoto Street in Madiun, A. Yani Street in Nganjuk, and Dhoho Street in Kediri. These streets are known nationally for the nasi pecel stalls where tourists from various places have been coming for decades to consume the nasi pecel, an iconic indigenous Javanese dish. Social psychologists have previously investigated the extent of inter-ethnic relations, accounts of the tensions, the political discourse, and the negative effects of the tensions on the communities [8,9]. Research investigating these topics has primarily been conducted using quantitative surveys and group-level analysis. Whilst such an approach and level of analysis has its own strengths, it omits detailed accounts regarding the complex and dynamic nature of the conduct of everyday life in these communities in which inter-ethnic relations are enacted [10].

The focus on the collaborative relations between Javanese and Chinese Indonesians in Indonesia is particularly important considering the history of relations between the two ethnic groups. Scholars have frequently depicted these relations within the frame of the often-tense histories between indigenous *(pribumi)* and non-indigenous ethnic groups, although recent research has also found that the tensions are now somewhat softened [11]. Although Chinese Indonesians have been present in Indonesia since the 14th century, they are generally still not perceived as part of Indonesia [12]. Chinese Indonesians, who make up less than 3 % of the 270 million total population of Indonesia, have often been involved in conflicts with “native” Indonesians. In the classic work on Javanese and Chinese Indonesian relations, scholars such as Carey [13] have documented the stereotypical assumption that there will be calamity whenever a Javanese forms an intimate relationship with a Chinese Indonesian partner. The tragic death of Prince of Mangkunegaran, Pangeran Harya Radityo Prabukusuma, is said to be his fault after the he had intimate relations with a local Chinese Peranakan girl [13].

Moving forward, the 1965 genocide grew out of attacks on alleged members of the Indonesian Communist Party which broadened to attacks on Chinese Indonesians for their association with the communism of (mainland) China, and Chinese Indonesians in various cities in Indonesia were attacked. In 1966, the Government of Indonesia adopted a policy of forced assimilation, in which many Chinese Indonesians were forced to change their Chinese names to Indonesian names. The more recent 1998 anti-Chinese violence in various big cities in Indonesia, in which many kiosks and houses were targeted and attacked by local demonstrators, has been a traumatic life event for many Chinese Indonesians [14]. Until today, in some places, such as Yogyakarta, the Government of Indonesia regulates the ownership of land by Chinese Indonesians. This series of events provides evidence of how inter-ethnic relations between Javanese and Chinese Indonesians are often seen adversarial [1].

However, we also acknowledge that inter-ethnic relations are complex and examples of collaboration between Chinese Indonesian and Javanese have also been documented. For example, scholars have documented that when early Chinese migrants arrived in some areas of Java, there was collaboration between the immigrants and local people who assimilated some of their food and cultural practices [15]. However, the accounts of these collaborations have generally been written from a historian's point of view and accounts of lived experiences are limited. Other scholars have also documented how some entrepreneurs during the Dutch colonial era developed joint ventures involving Javanese aristocrats and Chinese Indonesian entrepreneurs [13]. However, this collaboration only involved aristocrats and not Javanese commoners. Furthermore, accounts of how these collaborations were enacted are also limited [2]. We have sought to fill this gap by observing these types of collaboration more closely. In doing so, we needed a theoretical framework that could help us to explore the everyday conduct of inter-ethnic collaboration.

In conceptualising our study, we used the concept of the conduct of everyday life [10] and social practice theory [16], which enabled us to explore the connections between the *local* (micro) levels which are experienced by local nasi pecel sellers and the *general* (macro) levels of society [17]. Our understanding was informed by social philosophers such as Georg Simmel [18], who have argued that the general (macro) is reproduced through the particular (micro), especially within specific spatially located interactions. These theories overlap in the field of social psychology and have been influential in informing discussions on the mechanisms by which people demonstrate agency in creating their lives and, in doing so, reproduce their cultural traditions.

Following the key theorists of the everyday [10,18], we believe that everyday life is more than just routines and disruptions. Everyday life serves as a site where people interact with others from various backgrounds [19]. The concept of everyday life is particularly important for informing discussion of how our everyday activities operate at both the personal and the collective levels. The key assertion of the psychology of the conduct of everyday life is that ways of being contribute to the reproduction of broader social structures in society [20]. Previous scholars have also used the concept of the conduct of everyday life to study inter-ethnic relations in various settings. For example, everyday cooking practices can be vital for partners in inter-ethnic marriages, where learning to cook a partner's traditional food can be a way to show affection to a partner from a different ethnic group [5]. In this research, we have extended this scholarship to the study of how such inter-ethnic relationships are reproduced through the practice of selling traditional food in East Java. Specifically, we have approached the everyday activities of selling nasi pecel as a site for the reproduction of inter-group friendship.

Social practice theory has also been important in research into the conduct of everyday life and provides an explanation for how our everyday activities provide inter-cultural spaces for people from different backgrounds [16]. The key assertion of this theoretical framework is that our everyday lives are produced through mundane performative activities and reproduce a dynamic web of cultural expectations. As such, our everyday activities, which involve the use of objects, have implications for culture. For example, prior study documented that the use of everyday objects such as onion peeling machine can be important for showing affection and for the reproduction of the broader collaborative efforts between Javanese wife and Chinese Indonesian husband in generating income for their household [5]. Previously, scholarship based on social practice theory has explored the practices around food, including traditional food. For example, scholars have found that the practices around food are associated with relational ties, heritage, identity, and class [21]. What remains unclear is how everyday processes around food can reproduce inter-ethnic relations.

This paper documents the lived experiences of inter-group friendship within the context of two ethnic groups with a history of tensions, focusing on the everyday practices of selling nasi pecel. Specifically, we have moved beyond the literature that has generally framed the practice of selling nasi pecel as a purely entrepreneurial practice, as addressed in the following section. Specifically, we draw attention to how class and socio-economic status textures the everyday friendships between Javanese nasi pecel sellers and Chinese landowners, as exemplified in the practice of selling nasi pecel. In the next section, we provide a brief account of nasi pecel as a traditional food for indigenous people in East Java. We then discuss the current scholarship of food-related practices, which has shifted to viewing the selling food as more than just an entrepreneurial activity.

### 1.1 A brief history of nasi pecel

**Fig. 1:**
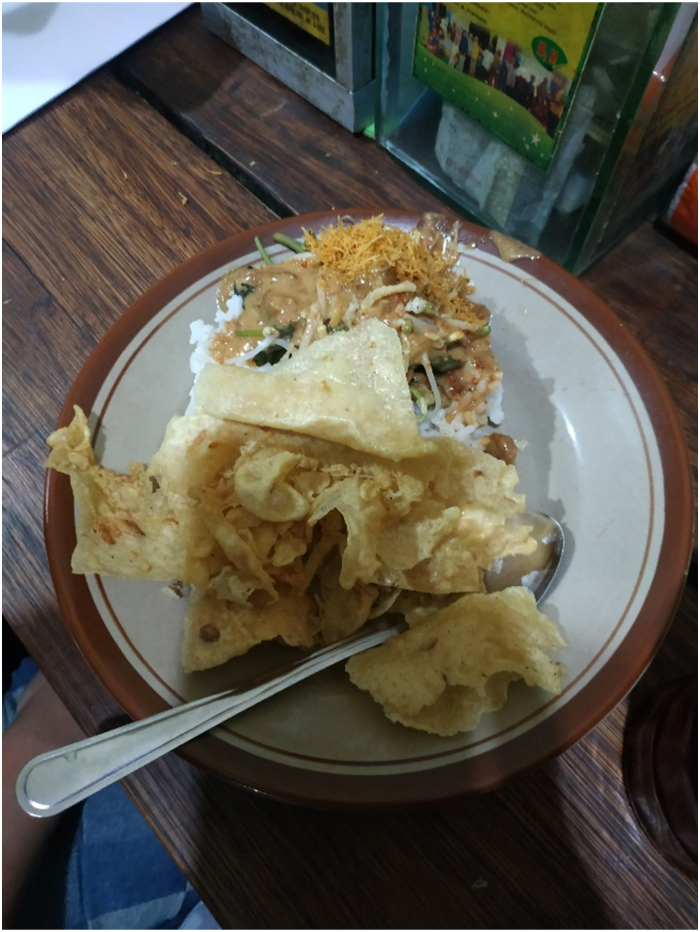
A plate of nasi pecel.

Nasi pecel is a traditional food for most people in Java (see Fig. 1). Mostly consumed in the morning and evening, nasi pecel is commonly found in Mataraman cities such as Madiun, Nganjuk, Kediri, Jombang, Blitar, Tulungagung, and Ponorogo. In these cities, nasi pecel has been held up as a signature cuisine by local governments developing a city's branding as a “nasi pecel city.” Each city has different recipes as the vegetables served in nasi pecel vary according to seasonal availability, but generally, a plate of nasi pecel consists of rice, vegetables such as boiled bean sprout and cassava leaves, peanut sauce, and crispy *rempeyek* (deep-fried savoury Javanese crackers) [22]. Some sellers include additional protein, such as fried chicken, chicken hearts or lungs, fried or scrambled eggs, and tofu or tempeh. Generally, Nasi pecel is described as a vegetable salad [23]. The price of nasi pecel varies from 5000 rupiahs (USD 0.32) up to 15,000 (USD 1), depending on whether the nasi pecel is sold by individual street vendors or in a restaurant. Recently, nasi pecel has also been found in many other parts in Indonesia and is also available in online shops for deliveries.

Historically, descriptions of nasi pecel date back to 1800 AD and it appears in the classic Javanese books, *Serat Centhini* and *Babat Tanah Jawi* [24]*.* Nasi pecel features in Javanese folklore as a dish for special occasions [25]. In the pre-Independence era, nasi pecel was initially known as the special dish of the Javanese royal family, which was then developed as a dish for common people in Java. Today, nasi pecel is an identity marker for many Javanese families. Whilst many people regard nasi pecel as central to their cultural identity as Javanese, interestingly, many people in Java describe the peanuts in the peanut sauce as Chinese peanuts *(kacang Cina)* [25]. Peanuts were brought to Indonesia by Chinese immigrants to Indonesia in the 14th century [24]. Therefore, many anthropologists have argued that nasi pecel is a dish that incorporates Javanese and Chinese cultures [24].

Nasi pecel has been studied widely in Indonesia from various perspectives. Whilst the studies from the perspectives of gastronomy and nutrition are extensive [22,23,25], scholars have also found that nasi pecel is more than just food. Scholars, for example, have investigated national politicians’ use of nasi pecel as a subject of political negotiations [26], and to frame themselves as humble and close to the people. In this paper, we have sought to extend the view of nasi pecel as more than just food and the selling of nasi pecel beyond an entrepreneurial activity by approaching the practice of selling nasi pecel as a site for the reproduction of inter-ethnic friendship. In doing so, we were interested in exploring how the seemingly mundane practices behind preparing and selling nasi pecel by Javanese nasi pecel sellers are vital in the reproduction of inter-ethnic relations.

## 2 Selling nasi pecel: Beyond an entrepreneurial activity

Traditional food such as nasi pecel is an important constituent in urban and rural identities globally and has become an object of attraction for tourists. Scholars from various disciplines have invested extensive effort into researching traditional food [27,28]. However, the approaches to such research have been predominantly focused on issues of scarcity, obesity, and nutrition [29]. Many of the investigations into the consumption of traditional food have considered dietary practices, which traditional food is “right to eat,” the individual predispositions of people interested in traditional food, and the scarcity of such food [30]. Such research has predominantly focused on individual-level analysis to investigate behaviours around eating traditional food. Subsequently, in the literature, traditional food is often reduced to a tourism element or fuel for health [27]. Although the perspectives of tourism and nutrition offer important avenues for exploring traditional food, theorists such as Claude Levi Strauss have long discussed how food is also implicated in the reproduction of culture [31].

Relatedly, recent scholarship has documented how traditional food has much more to offer than just as fuel for our bodies. It also has significance in relation to place identity, well-being, and collective memory, as well as offering a space for inter-group interactions [21]. Traditional food such as nasi pecel is also often associated with the cultural identity of a particular indigenous ethnic community [28]. Subsequently, scholars have begun to conduct reviews to map the traditional food practices in many countries and there has been a proliferation of efforts to understand how we can present traditional food in the current era [32]. These includes efforts to create innovative variations on traditional food products which benefit from concepts and theories of entrepreneurship [33]. This scholarship serves as a strong base showing how nasi pecel can be instrumental in shaping the identities of cities and communities.

Whilst the study of traditional food has great importance, the social practices surrounding traditional foods are also foundational for many societies. As in many countries in Asia, the practice of eating traditional food such as *ketupat* (rice cake) during Eid is an important reason for families in Indonesia to gather with broader familial networks [34]. Eating ketupat with family members offers a crucial space for many families to communicate cultural values to the younger generations. For example, whilst eating the food, elders in Malaysia can talk about their concerns with their children and grandchildren, including the need to preserve traditional food by continuing the everyday practices of cooking and having meals together [32]. This scholarship resonates with philosopher Georg Simmel's explanation of the family meal as a site that interweaves material objects and sociability [18]. It is through having meals and eating nasi pecel together that many families in Java produce and reproduce the cultural identities of both the families and the cities.

Previously, many scholars have focused on the financial issues, such as returns on investment for producers, around selling the traditional food such as nasi pecel which contributes to the branding of cities [35]. This scholarship is important for informing the practice of selling nasi pecel for daily consumption by locals and as a tourist attraction. Scholars in Canada have found that selling traditional food involves complex adjustments, such as creating new ways of cooking and presenting the traditional food to accommodate the tastes of visitors [36]. What is so far underdeveloped in this scholarship is investigation into the role of the practice of selling traditional food such as nasi pecel in producing and reproducing inter-ethnic relations, which we directly address in this paper.

## 3 The research approach

This article draws on findings from a 2-year research project investigating the role of the practice of selling traditional food in shaping the cultural identities of cities in East Java. This study was approved by the ethics committee of Universitas Nusa Tenggara, Indonesia (Ethics Number: No 2023314-KEPK). The fieldwork was conducted over an eight-week period and used verbal, observational, and visual, qualitative methods to document the everyday lives of nasi pecel sellers in four cities in the Mataraman district of East Java: Madiun (a population of over 765,135), Nganjuk (1,178,972), Jombang (1,393,813), and Kediri (1,673, 158). These cities were relevant in this study because they are known nationally as the cities of nasi pecel, where many locals and tourists consume nasi pecel every day. The four cities are neighboring cities within 1–2 h’ drive away from each other. Demographically, Chinese Indonesian is the minority ethnic group in these four cities and constitutes approximately 0.1 % of the population. Among four cities, Jombang is the city with the highest number of Chinese Indonesian population, constituting around 13,938 people, following with Kediri (13,765), Nganjuk (11,896) and Madiun (78,625) [37].

We included both morning and evening nasi pecel sellers to provide a more holistic understanding of the everyday lives of the sellers. During the fieldwork, we used a snowball technique to identify prospective participants across the four cities. The 30 nasi pecel sellers recruited in the four cities had various backgrounds (see Table 1 in the Appendix for a summary). As we can see in the table, of the 30 nasi pecel sellers, 9 of them sell nasi pecel in Nganjuk (30 %), 8 in Jombang (26.7 %), 6 in Kediri (20 %), and 7 in Madiun (23.3 %). Twenty of the 24 sell nasi pecel at night (66.6 %), 5 sell in the morning (16.7 %), and 5 keep their stalls open 24 h (16.7 %). They have been selling for periods from 5 months to 20 years. All participants participated voluntarily and received a small amount of compensation. They all provided their written consent to participate in this study.

To interview the 30 nasi pecel sellers, we employed two techniques: go-alongs and photo-elicitation interviews. This go-along method [38] enabled us to generate stories around the practices of selling without disrupting the participants' routines. A central feature of this approach was our immersion with the local sellers in these cities, where we bought nasi pecel and interacted with the sellers and the customers to experience the natural exchanges. As time went on, we got to know the nasi pecel sellers and invited them to take part in the research. We then asked for biographical information to understand how long they have been selling nasi pecel, who cooks the food, their relationship with the owner of the site, and their everyday lives of preparing and selling nasi pecel. The conversations were primarily conducted in the Javanese language, which is the language Javanese people typically use in everyday life. The main challenge in this research was our participants' perceptions of the practice of selling as routine and something to be taken for granted. Secondly, we also conducted photo-elicitation interviews [39] to explore snippets of their everyday lives. The nasi pecel sellers were asked to photograph the mundane aspects of their lives that spoke directly to the practice of selling nasi pecel. The photographs could focus on anything, such as the people, activities, objects, or places that were associated with their everyday practice of selling nasi pecel. In the next visit, we discussed the significance of those photographs. These methods allowed us to document the ways in which traditions and practices around traditional food are enacted in everyday life from the sellers’ points of view [39]. All participants provided written consent for the photographs taken to be included in the manuscript for publication.

To make sense of the empirical materials generated, we utilized the power of the case study [40]. Our engagements with the 30 nasi pecel sellers in the four Mataraman cities have enabled us to access a large body of empirical materials comprising 35 interviews and over 200 photographs. The case-comparative method was particularly useful for constructing the stories from each of the sellers as cases for considering the traditional food-related practices and the broader landscapes within which the practices of selling were situated. Given the size and the complexity of the research corpus, in the following section we discuss the stories from six nasi pecel sellers, Amy, Nin, Maya, Tin, Sony, and Sari (pseudonyms), which resonated with those of the other 24 participants. The six participants were aged 22–74-year-old when we conducted the study. These nasi pecel sellers were selected because their stories of their everyday practices of selling nasi pecel were strongly associated with the Chinese Indonesian landowners. The chosen participants’ narrations of their relationality with the Chinese landowners are important because in the literature relationships between Javanese and Chinese Indonesians are often seen as conflictual and segregated [1].

As our analytical strategy to make sense of these complex empirical materials, we followed impressionistic orientation towards visual inquiry into the conduct of everyday life [41]. This approach has been applied extensively in various contexts, such as the everyday life of homeless, everyday inter-ethnic marriages, and everyday migrant practices [5,17,21]. In conducting impressionistic approach, we acted as *bricoleurs* [42] by working in an interdisciplinary way to generate insights into the practices of selling food. The first author initiated the analytical process by examining all the interview transcripts, observation reports, and photographs to generate the main themes and additional themes that featured strongly in each city. In this phase, we identified that the narratives on the everyday collaborations between the Javanese nasi pecel sellers and Chinese landowners consistently emerged in the stories of 30 nasi pecel sellers in these four cities. Then, the first author asked the second and third authors, who were also involved in the field work, to provide specific examples for each theme. We also asked our five research assistants who helped us during the process of gathering empirical materials to provide specific examples which emerged from their engagements with the participants. The stories from the six sellers were selected because of the depth of their stories. Whilst the remaining 24 nasi pecel sellers also mentioned the collaborations with the Chinese landowners, most of them were not extensively narrated. We then worked abductively [43] to read, re-read, and dialogue those examples with the related literature to deepen our understanding of each case. All examples, including the associated photographs, were then collated and analysed to inform further discussions in relation to the scholarship of the conduct of everyday life and social practice theory and to deepen our understanding about each case.

## 4 Relationality with the Chinese shop owners

The everyday life of a nasi pecel seller is inseparable from the process of preparing a place to sell the nasi pecel. Our participants demonstrated that central in the process of such preparation are their relationships with the Chinese Indonesians who own the shops. In this section, we discuss how the relationality between the Chinese Indonesian landowners and the Javanese nasi pecel sellers is reproduced through the everyday practices of talking, telling jokes, and gifting. Such seemingly mundane practices are important in enacting the collaborative spirit between the entrepreneurs [20]. As we demonstrate through the three exemplars below, the practice of selling nasi pecel can be seen as a site of inter-ethnic friendship between the Javanese and Chinese Indonesian entrepreneurs.

In the city of Madiun, nasi pecel is known as the main traditional food and it has become famous internationally. Many nasi pecel sellers occupy sites on Cokroaminoto Street where they offer nasi pecel to customers. One of the nasi pecel sellers in Madiun, Amy, talked at length about how she engages with the Chinese Indonesian owner of the space where she has her stall. In doing so, Amy used the words *dolan* (visit) and *jagongan* (conversation), showing how relationality with the Chinese Indonesian owner is emplaced within the particular practices of dolan and jagongan*.* For her, visiting and conversing about various things with the Chinese Indonesian owner has helped her to create a friendship with him. Amy often visits the Chinese Indonesian shop owner around 30 min before she opens to converse and “say hi.” Dolan and jagongan are practices she observes as “something that you must do” to gain respect. This reflects the Javanese practice of *kulanuwun* which refers to the ethics of asking permission of the owner prior to doing anything. Amy explained:

I came here 30 minutes early to visit him in the shop. To say hi to him and his family. To check if he and his family are doing alright. My business is all about earning money for survival. But they kindly gave this space to me for free. So, it would be appropriate that I acknowledge their generosity by visiting them. Sometimes I invite them to eat my nasi pecel and talk. This is my way to *kulanuwun.* To ask permission.

This story reflects that the Javanese seller's relationality with the Chinese Indonesian owner goes beyond the practice of selling nasi pecel itself. For the Javanese seller, the practice of selling nasi pecel is textured through the art of maintaining relationship with the owner through the Javanese concept of *kulanuwun*. This shows that indigenous cultural values play an important role in informing her understanding about what it means to be a good Javanese nasi pecel seller. In practicing *dolan* and *jagongan,* the Javanese seller is positioning herself as a Javanese entering Chinese Indonesian space and coloring that space with Javanese wisdom. Her practice of coming 30 min early is a way of enacting respect as a sign of her gratitude for using the place for selling and earning her living [6,16]. Here, the Javanese seller demonstrates that the practice of selling nasi pecel goes beyond a purely entrepreneurial practice. She colors the city with her inter-cultural friendship with the Chinese Indonesians.

**Fig. 2:**
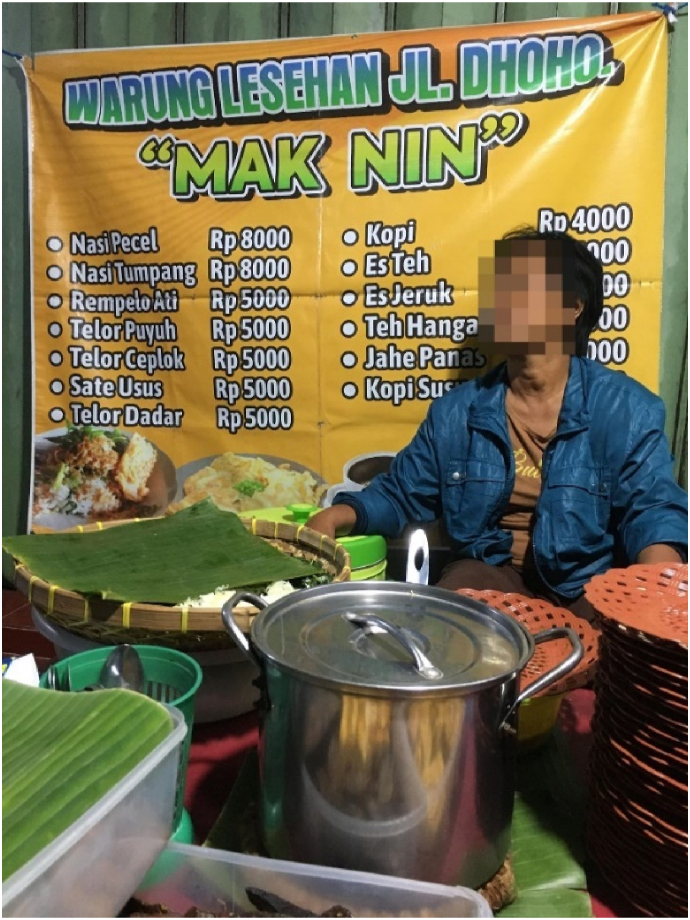
A space in front of a Chinese-owned motorcycle spare part shop.

In Dhoho Street in the city of Kediri, there is a similar pattern, with Javanese nasi pecel sellers occupying the space in front of Chinese Indonesian-owned shops. Nin (pseudonym), a Javanese seller, seconded the importance of having good relationships with the Chinese Indonesian shop owners. She particularly highlighted the centrality of jokes in establishing their relationships. Nin reflected on how jokes have been useful for her to make sure that they “still like” her to use their space to sell nasi pecel. As she uses the space for free, she understands that the owner could ask her to move somewhere else. Here, she uses jokes as an instrument to help her navigate potential tensions with the Chinese Indonesian owner. Nin further explained:

We have been here for years selling nasi pecel and I am so happy to get this space (see Fig. 2) for free. It helps us to survive every day. This road is the main road in the city and many people from various cities come here for nasi pecel Kediri. However, getting permission to use this space for free also makes me anxious. I am afraid to make mistakes with the owner. Can you imagine, he can ask us to move somewhere else. But he is a good bloke. He often comes here for nasi pecel or coffee. We often exchange jokes. He said my coffee is not delicious. But he often comes here for coffee! I said, it is indeed not delicious, but I *suwuk* (bless) it for him to like it [laughter]. Every time we exchange jokes, I relax. He still likes me to be here.

For many sellers, a vital aspect of their efforts to enact the practices of selling nasi pecel is the relationality with the Chinese Indonesian owner that is produced and reproduced through exchanging jokes [17]. The Javanese seller's humor and jokes have been fundamental in maintaining her relationship with the Chinese Indonesian owner and creating a basis for her nasi pecel business. Their humor functions as a social glue and a defence against her insecurity and worry about being displaced. Here, the jokes blend their identities as entrepreneurs from different social classes, in the sense that the Javanese nasi pecel seller, who is from a low socio-economic background, and the more affluent Chinese Indonesian interact casually in everyday life. Previously, scholars have documented how individual entrepreneurs develop their own senses of humor to establish their social identities [44]. This includes the use of humor to shape and delegitimize stereotypes. We expand on this finding by situating the jokes as instruments for entrepreneurs from different ethnic groups and social classes to negotiate their relationships.

**Fig. 3:**
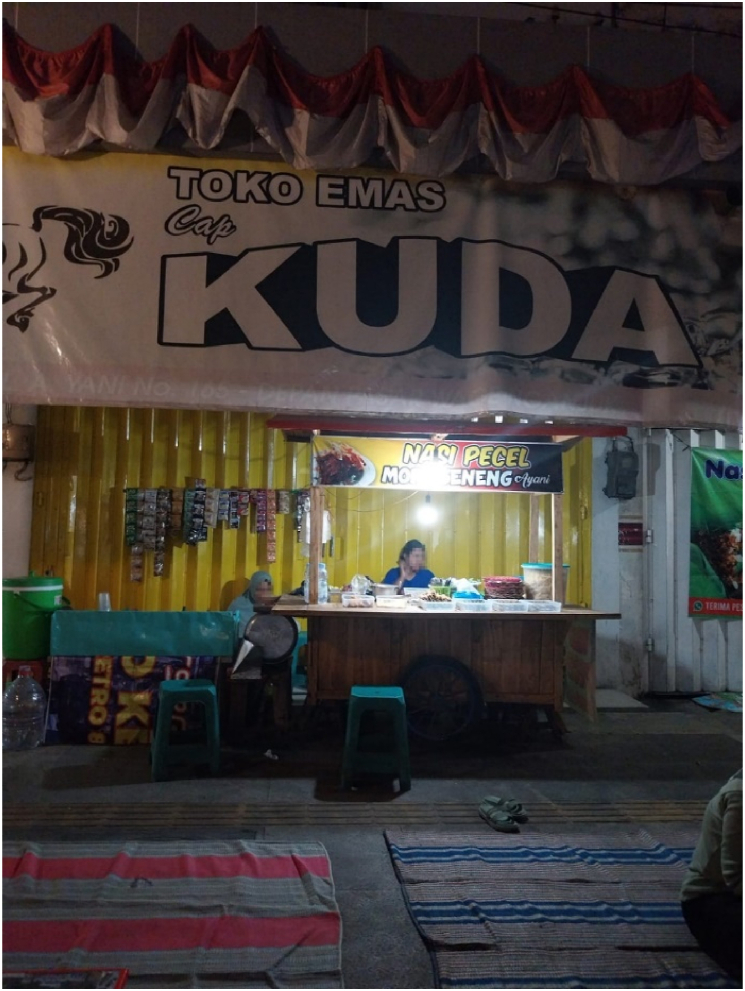
A space for selling nasi pecel in front of Chinese-owned gold shop.

Maya (pseudonym), another Javanese nasi pecel seller in Nganjuk, has been selling nasi pecel for 10 years in front of a gold shop in A. Yani Street in Nganjuk owned by a Chinese Indonesian entrepreneur. She originally comes from a remote district of Nganjuk and has lived in Nganjuk with her daughter for over 10 years. This move has given Maya and her daughter closer access to the city. Her husband, a truck driver, frequently travels back and forth between Jakarta and Nganjuk. During our engagements, she talked about how her practice of selling nasi pecel forms a collaborative space between her and the Chinese shop owner which allows her to get the free electricity which makes the practice of selling nasi pecel work. Her efforts in selling nasi pecel are inseparable from the negotiations with the Chinese Indonesian entrepreneur who kindly offered her the space in front of his gold shop for selling. Reflecting on the negotiations between her and the Chinese Indonesian entrepreneur, Maya talked about how the opportunity to use the space is hugely important for her to improve their lives. In doing so, she referred to how macro-policies such as the regional minimum wage in the city have had direct impacts on her tactics for selling nasi pecel by influencing her choice of a strategic site:

I am so grateful to meet with the owner of this shop. When I asked him for permission to use this space to sell my nasi pecel (see Fig. 3), he kindly gave me permission to use it. This is a really ideal place to sell, which I would never be able to buy with my own money. As you know, people in Nganjuk have low incomes and therefore might not be able to buy, let’s say not only a house or land, but also expensive nasi pecel! I totally get it and that is the reason why I sell my nasi pecel at 5,000 rupiahs (0.33 US dollars). But of course, I need to have a location where I can sell a lot of nasi pecel. I was fortunate to meet the Chinese owner, who allows me to use this space for free. I regularly visit his house simply to bring him and his family my nasi pecel to say thank you.

A. Yani Street in Nganjuk has been renowned for its night market for decades. The street, which is in a shopping area owned by Chinese Indonesians, is transformed by the nasi pecel stalls run by small-scale Javanese entrepreneurs. The reflection from the Javanese nasi pecel seller above describes how her status as a small-scale entrepreneur means she is unable to own a site in a place such as A. Yani Street. Mentioning the regional minimum wage rate in Nganjuk (around 2,000,000 rupiahs or 131 USD per month), she acknowledged that the low incomes only allow people to survive day-by-day and prevent them from owning land in the downtown area. The opportunity given to her by the Chinese Indonesian has allowed her to temporarily reclaim space in the city through the practice of selling nasi pecel, and in doing so, reproduce the identity of the city. The permission from the Chinese Indonesian, the space offered, and the relationality between them are important elements of the sustainability of her small business. The Javanese nasi pecel seller's gifts of nasi pecel to the owner's family are worthy of further reflection. She provided an account of the centrality of gifting in maintaining their relationality, emphasizing her relational obligation as a Javanese. Here, she reciprocated his kindness by providing a gift [45].

## 5 Relationality with the Chinese neighbors and broader Chinese communities

During our engagements, participants also shared that the practice of selling nasi pecel is also textured through the relationality not only with the Chinese owners, but also with the Chinese neighbors and the broader Chinese communities, through the everyday practices of accessing electricity and water and interacting socially. By situating the relationality of nasi pecel within the public realm, this section discusses how the practice of selling nasi pecel reproduces broader collaborative inter-ethnic interactions [17]. As we demonstrate throughout the section, central for our participants is maintaining harmony with all the parties who directly and indirectly contribute to what makes their businesses work. Beyond the economic transactions, we can see the cultural values underlying the relationality for our participants.

**Fig. 4:**
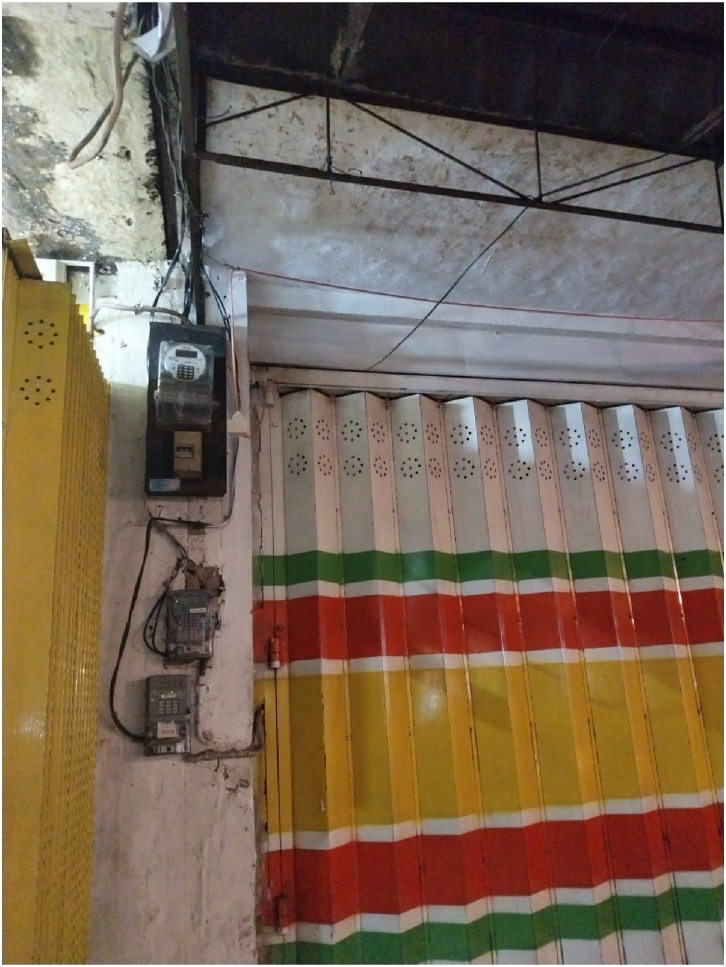
Setting up electricity from the Chinese Indonesian-owned shop.

During interviews, Tin (pseudonym), a Javanese nasi pecel seller, shared how she accesses free electricity from the community leader, Pak RT. She had only been running her nasi pecel business for the last 6 months. As she sells the nasi pecel at night, Tin acknowledged the need to access electricity for her *rombong* (stall). She asked the owner of the shop for help, and he then connected her to the community leader, a Chinese Indonesian. She talked animatedly about how the community leader helped her out by setting up the electricity every night. This experience gave her an understanding of the system of electricity use among Chinese Indonesian shop owners. Tin shared:

I meet Pak RT every day to set up electricity for my nasi pecel. He is so kind to allow me and other tenants to access the electricity for free. He regularly checks the cable (see Fig. 4) to make sure everything is fine. It is through Pak RT that I could get to know the neighbors here. As you know, people like me would never have the chance to befriend Chinese, if not for selling nasi pecel here.

The assertion above revealed how the Javanese seller positioned herself as an outsider who was not part of the Chinese community in the area [46]. However, the practice of selling nasi pecel in that space had made her informally part of the Chinese Indonesian community. Prior research on entrepreneurship has tended to frame the practice of selling simply as an entrepreneurial business practice involving strictly economic transactions [47]. However, her assertion provides an alternative view on how the practice of selling nasi pecel also involves the broader neighboring community. In the process of helping her, the community leader brought her and her business into the Chinese community there.

**Fig. 5:**
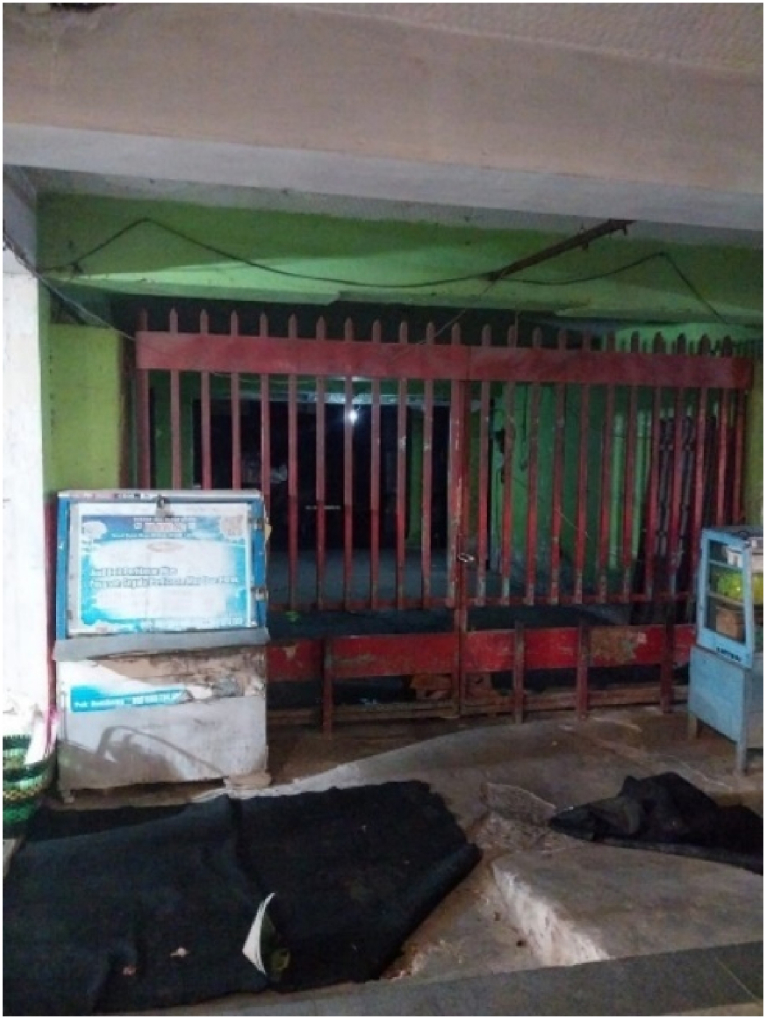
The house of the neighbor who helps nasi pecel sellers to access water.

Whilst electricity was a central element for our participants, also vital was access to water. Sony (pseudonym), a man who had been selling nasi pecel for 17 years, shared that he could sell nasi pecel because of the help from the Chinese neighbor who lives next to the shop where he sells the nasi pecel. As such, it was not only the Chinese Indonesian owner, or a community leader as the previous seller described, but also the neighbor who helped him. The access to water has been instrumental in shaping the relationality between him, the owner of the shop, and the neighbor. As such, it is the practice of selling nasi pecel that enables him to develop relationships with the neighboring Chinese community in the city. Sony explained:

The one who gives me electricity is different to the one who gives me water. It is the neighbor who lives over there (see Fig. 5). He gives me water for dishwashing. I asked him if he wanted me to pay, but he refused. He wants me to succeed and that is really kind of him. I come from a district where the population is predominantly Javanese. People often talk about Chinese, and they can have their own opinion. But for me, after spending nearly a decade in this place, I can safely say that Chinese Indonesians are the best. They have been helping me to earn money to live.

The quote above provides an exemplar for how the relationality with the Chinese Indonesian community is again reproduced through the mundane practice of accessing water [19]. Here, he provides evidence of how the practice of selling nasi pecel has enabled him to move from the position of an outsider who never interacts with Chinese Indonesians into the position of an insider who interacts intensively with Chinese Indonesians. Further, he described how, through everyday interactions with the neighbor, he could understand the value of the inter-cultural friendship, which challenged his initial perceptions of Chinese Indonesians [11]. Previous research into relations between Javanese and Chinese Indonesian entrepreneurs has paid limited attention to collaborations in everyday life [1,13]. This exemplar directly fills that gap.

**Fig. 6:**
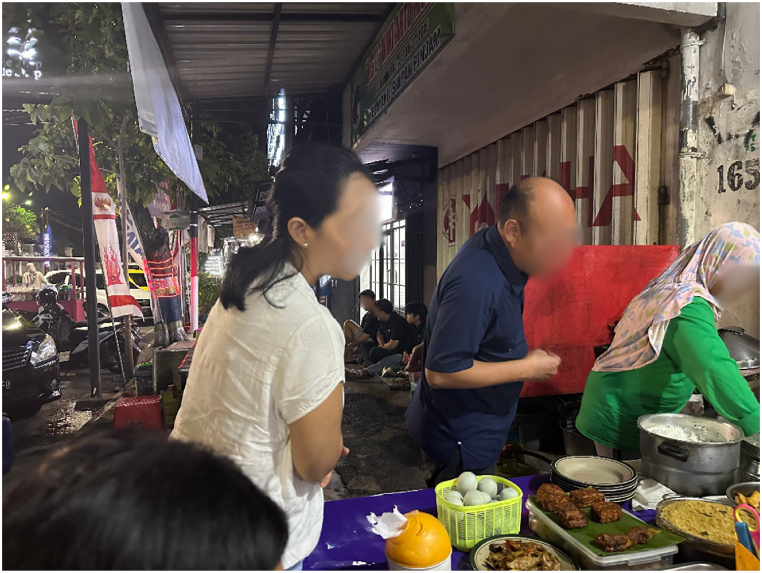
Chinese Indonesian church members buying nasi pecel.

Our participants not only talked about the neighboring Chinese community around the main street but also provided evidence of how Chinese Indonesian owners helped the participants by bringing their broader Chinese communities to come and eat nasi pecel together. As Sari (pseudonym), a Javanese seller in Madiun explained, the shop owner, a Christian Chinese Indonesian, often invited his church community members, who were predominantly Chinese, to eat together in her place. Sari asserted:

What I like about the shop owner is he often invites his church friends (see Fig. 6), most of them Chinese, to eat together in my place. As you know, I have so many customers every Sunday. They are dressed up, just after the church service, and eating together. Wow. Suddenly, my nasi pecel place is so busy. That is how he helps me, through word of mouth. That is how people know my nasi pecel. Because each of them will tell their own relatives. I now have many Chinese customers. I believe that that is because of him.

Sari provided an exemplar for how the relationality with the Chinese Indonesian owner and neighboring community has been extended to include the broader context of the Chinese community in faith-based institutions such as churches [6]. Her stories provided insights into how relationality is not fixed but rather develops over time and involves wider relationalities and multiple spatialities. What she is doing might only be local and just part of other enterprise in that street, however it does not mean that what she is doing is insignificant. Sari has demonstrated that her nasi pecel has been influential in creating a public space where many Chinese Indonesian people come and mingle and interact with others from different ethnicities and religions. Her nasi pecel stall has been serving as an encounter space for people from various backgrounds to casually converse on various issues [7,48].

## 6 Discussion

In the literature, inter-ethnic relations between Chinese Indonesians and Javanese have generally been presented as adversarial [1,11,13]. Assumptions about inter-ethnic relations between Chinese Indonesians and Javanese are often framed within the context of indigenous and non-indigenous and are predominantly discussed within the context of inter-ethnic relations in national-level politics. Chinese Indonesians are often seen as non-*pribumi* and they have experienced various form of discrimination in everyday life, continuing even to the present day. The notion of *pribumi-*ism, which is often associated with Javanese Muslims, sees them as different to Chinese Indonesian people, who are predominantly Christian and Confucian. In this article, we have provided empirical evidence of collaborative entrepreneurial relationships between Javanese nasi pecel sellers and Chinese Indonesian shop owners [[5], [6], [7]]. Whilst Chinese and non-Chinese Indonesians are often perceived as divided politically [1], our findings suggest that there are commonalities and solidarity, which are enacted through the everyday practices of selling nasi pecel.

Alongside the issues of business development, consumer behaviour, and nutrition, the reproduction of inter-ethnic solidarity is also vital for our participants in their everyday practices of preparing and selling food. As we have demonstrated throughout the article, these everyday practices involve intense cooperation and negotiation between the sellers, who are predominantly Javanese, and the Chinese Indonesian owners of the sites. The collaborative nature of the relationships between both sides as entrepreneurs is particularly important to reflect on, as studies into entrepreneurship predominantly discuss competitiveness, benefits and advantages, the added value of the business, and other entrepreneurial elements that assert a zero-sum game [47]. Our participants provided some exemplars for collaborations that can exist between Javanese micro-scale entrepreneurs and Chinese meso-scale entrepreneurs. The practice of collaboration has produced and reproduced a space for the Javanese micro-scale entrepreneurs to write the identity of the city through their nasi pecel.

The practice of selling traditional food has been important in many cities in Indonesia as a tourist attraction and an entrepreneurial activity. Such practice has proven significant in generating income for micro-scale entrepreneurs and in developing the cultural identities of the cities [47]. Mainstream research into traditional urban gastronomy has tended to focus on this practice within the context of business development, consumer behaviour, and nutrition, and has overlooked the seemingly mundane activities that shape the routines and practice of selling traditional food [30,33,49]. Drawing from the scholarship of social practice theory and psychology and the conduct of everyday life [10,16], we have extended the scholarship on traditional urban gastronomy by looking at the everyday practices of selling nasi pecel in four cities in Indonesia. As we have demonstrated, the routines of selling nasi pecel are not just about business development, consumer behaviour, and nutrition, but also involve ethnic identity and inter-ethnic cooperation.

Our investigation contributes to the scholarship on everyday life and social practice theory by extending the concepts to document inter-ethnic relations and its intersectionality with traditional food and the use of space in the city [17]. Previously, scholarship on social practice theory has provided limited account on how the concept of practice can explain inter-ethnic relations [6]. Literature on inter-ethnic relations from the lens of social practice theory generally referred that ethnic communities often try to pursue cultural originality, traditional lifestyle, and associated social practices by practicing a territorial separation, which characterizes a particular area with a particular ethnic group [50,51]. This notion of territorial separation also applies to the context of inter-ethnic relations between Chinese Indonesian and *pribumi.* Normatively, many Chinese Indonesians in Indonesia live in gated communities and Chinese-owned commercial areas as a way to build a sense of community with the ingroup and as a space of exclusion with the outgroup [52]. Extending such work, our findings documented how everyday practices of selling nasi pecel, which mostly is emplaced in the Chinese space, are crucial to weave such strict territorial separation. The everyday practice of selling nasi pecel is proven vital to enact, develop, and reproduce inter-ethnic relations in the city. The seemingly mundane activities that occurred in the Chinese spaces such as exchanging jokes, gifting, and accessing water and electricity have proven influential for the Javanese nasi pecel vendors to nurture the relationship between the Javanese nasi pecel sellers and the Chinese Indonesian landowners. In other words, the development of city branding of these cities as ‘the nasi pecel city’ is not an exclusive effort of the Javanese nasi pecel vendors. It instead involves the collaborative efforts of the Javanese nasi pecel sellers, the Chinese Indonesian landowners, the Chinese neighbors, the broader Chinese communities, and the consumers from the various ethnic groups in these cities. These elements are interwoven in the everyday practice of selling nasi pecel, which now serves as a site of inter-ethnic relations and solidarity in these cities.

We understand that for some readers, our findings might be affronting because of the prolonged historical tensions between Javanese and Chinese Indonesians [1]. As scholars have found, the tensions are now somehow softened [11]. We believe that the present focus on the everyday inter-ethnic collaborations is timely to provide a “common ground” for the ethnic groups by highlighting their efforts to learn from each other, adapt to and accommodate their cultural differences, and focus on the points of commonality. Central to our findings is the insight that the ways our participants find to collaborate to enact the practice of selling nasi pecel every day reproduce the identities of the cities as “cities of nasi pecel.”

Further research is required to explore this phenomenon, specifically from the perspective of the Chinese Indonesian landowners. The next phase of research should also involve the Chinese communities, such as the Pak RT (the neighbourhood community leaders), the leaders of nasi pecel sellers’ association, and the costumers to provide a more holistic framework.

## Data availability statement

The data that support the findings of this study are available from the corresponding author, upon reasonable request.

## CRediT authorship contribution statement

**Jony Eko Yulianto:** Writing – review & editing, Writing – original draft, Funding acquisition, Formal analysis, Conceptualization. **Gabriela Laras Dewi Swastika:** Writing – original draft, Resources, Methodology, Investigation, Funding acquisition, Formal analysis. **Indra Yohanes Kiling:** Writing – review & editing, Methodology, Funding acquisition, Formal analysis, Conceptualization.

## Declaration of competing interest

The authors declare that they have no known competing financial interests or personal relationships that could have appeared to influence the work reported in this paper.

## Figures and Tables

**Table 1:** The participants in the study

No	Pseudonym	City	Experience of selling nasi pecel	Selling time
1	Mursini	Nganjuk	14 years	Morning (06:00–10:00)
2	Sony	Nganjuk	17 years	Night (17:00–24:00)
3	Endang	Nganjuk	4 years	Night (17:30–24:00)
4	Dian	Nganjuk	6 years	Night (17:00–24:00)
5	Maya	Nganjuk	10 years	Night (18:00–24:00)
6	Ramlah	Nganjuk	8 years	Night (17:00–24:00)
7	Tin	Nganjuk	6 months	Night (17:00–24:00)
8	Petrus	Nganjuk	8 years	Morning (5:30–09:00)
9	Sriyah	Nganjuk	6 years	Morning (5:30–10:00)
10	Iman	Jombang	7 years	Night (17:00–02:00)
11	Ifa	Jombang	6 years	Morning (5:30–10:00)
12	Tri	Jombang	7 years	Morning (6:00–10:00)
13	Aseh	Jombang	15 years	Night (17:00–02:00)
14	Yoyon	Jombang	5 years	Night (17:00–23:00)
15	Khusnul	Jombang	6 years	Night (17:30–24:00)
16	Djiyah	Jombang	20 years	All day (06:00–24:00)
17	Marmi	Jombang	5 years	Night (17:30–24:00)
18	Parti	Kediri	6 years	Night (17:00–24:00)
19	Ani	Kediri	3 years	Night (17:30–24:00)
20	Nin	Kediri	7 months	Night (17:30–03:00)
21	Yul	Kediri	5 years	Night (17:00–24:00)
22	Sumi	Kediri	4 years	Night (17:00–24:00)
23	Siti	Kediri	2 years	Night (17:00–24:00)
24	Edy	Madiun	6 years	Night (17:30–03:00)
25	Gemi	Madiun	15 years	All day (06:00–24:00)
26	Amy	Madiun	5 years	Night (17:00–24:00)
27	Wir	Madiun	17 years	All day (06:00–24:00)
28	Sri	Madiun	15 years	All day (06:00–24:00)
29	Elang	Madiun	6 years	All day (06:00–24:00)
30	Sari	Madiun	5 months	Night (17:00–24:00)
